# Slm35 links mitochondrial stress response and longevity through TOR signaling pathway

**DOI:** 10.18632/aging.101093

**Published:** 2016-12-02

**Authors:** L. Aguilar-Lopez Jose, Raymond Laboy, Jaimes-Miranda Fabiola, Erika Garay, DeLuna Alexander, Soledad Funes

**Affiliations:** ^1^ Departamento de Genética Molecular, Instituto de Fisiología Celular, Universidad Nacional Autónoma de México, Cd.Mx. 04510, Mexico; ^2^ Unidad de Genómica Avanzada (Langebio), Centro de Investigación y de Estudios Avanzados del IPN, Irapuato, Guanajuato 36821, Mexico

**Keywords:** mitochondria, mitophagy, catalase, lifespan regulation, mitochondrial morphology

## Abstract

In most eukaryotic cells mitochondria are essential organelles involved in a great variety of cellular functions. One of the physiological processes linked to mitochondria is aging, a gradual process of damage accumulation that eventually promotes cell death. Aging depends on a balance between mitochondrial biogenesis, function and degradation. It has been previously shown that Tor1, Sch9 and Ras2 are activated in response to nutrient availability and regulate cell growth and division. A deficiency in any of these genes promotes lifespan extension and cell protection during oxidative and heat shock stress. In this work we report that in *Saccharomyces cerevisiae*, the uncharacterized mitochondrial protein Slm35 is functionally linked with the TOR signaling pathway. A Δ*tor1*Δ*slm35* strain shows a severe decrease in lifespan and is unable to contend with oxidative and heat shock stresses. Specifically, this mutant shows decreased catalase activity indicating a misregulation of ROS scavenging mechanisms. In this study we show that Slm35 is also relevant for mitochondrial network dynamics and mitophagy. The results presented here suggest that Slm35 plays an important role connecting mitochondrial function with cytosolic responses and cell adaptation to stress and aging.

## INTRODUCTION

It is now well acknowledged that mitochondrial function is compromised in aged cells and tissues [1]. For many years, the mitochondrial free-radical theory of aging was the prevailing explanation for the process of aging in living organisms [2]. This theory postulates that aging is the result of an accumulation of reactive oxygen species (ROS) originating from the mitochondria due to a homeostatic imbalance between oxidative stress and stress defense within the cell. Oxidative insults to mitochondria alter the mitochondrial DNA (mtDNA) inducing malfunctions of the respiratory chain complexes and thereby increasing the ROS production [3]. This creates a vicious cycle in which oxidative damage impairs mitochondrial function, hence mitochondria produce more ROS, which in turn promotes more damage, eventually leading to cell aging and death [4]. However, there is experimental evidence suggesting that ROS can also function as anti-aging molecules [5, 6] or as signaling molecules playing a crucial role in several processes that affect aging [7]. In addition to ROS, several other aspects of mitochondrial metabolism have been linked to aging: dietary restriction reduces oxidative damage and improves mitochondrial functions extending lifespan [8]; imbalance in mitochondrial dynamics contributes to oxidative stress and cell death during aging [3]; and mitophagy and mitochondrial biogenesis allow cells to adjust their mitochondrial content during aging [9].

In model organisms such as yeast, nematodes and flies, an extension of expected lifespan can be achieved either by a decreased food intake without malnutrition (dietary restriction) or by genetic alterations involving nutrient-sensing pathways [10]. In the budding yeast *Saccharomyces cerevisiae*, dietary restriction can induce lifespan extension via two conserved routes: one is centered on the serine-threonine kinases Tor1 and Sch9 [11]; and the second depends on Ras2, adenylate cyclase and Protein Kinase A (PKA) [12]. The contribution of mitochondrial metabolism has been shown to be key for mediating longevity in both pathways [1, 6, 13]. The Target of Rapamycin (TOR) signaling pathway, one of the main nutrient-sensing pathways in yeast, has been linked to regulation of lifespan [11, 14, 15]. When TOR is active, processes such as ribosome biogenesis, protein synthesis and nutrient import are maintained [16]. Reduced TOR signaling induces a series of responses, including an increase in mitochondrial translation and the concomitant increased abundance of oxidative phosphorylation complexes per organelle. This stimulates oxygen consumption and diminishes ROS production during the stationary phase, limiting cellular damage and extending yeast chronological lifespan (CLS) [17] and replicative lifespan (RLS) [18]. Tor1 activates the effector Sch9, whose downregulation also extends lifespan [19]. The mammalian ortholog of Sch9, the ribosomal S6 kinase 1 (S6K1), influences lifespan and age-related pathologies [20]. The Ras/cAMP/PKA pathway has also been identified as a pro-aging pathway [21]. *RAS2* deletion increases glycogen accumulation, superoxide dismutase (SOD) and catalase activities, thermotolerance and chronological survival by two-fold [22]. Inhibition of both pathways converge in the activation of the stress resistance serine-threonine kinase Rim15 and its downstream transcription factors Msn2/4 and Gis1 [23, 24]. These transcription factors enhance cellular stress responses through heat shock proteins and antioxidant enzymes, leading to lifespan extension [25].

In addition to the processes described above, the TOR pathway controls autophagy, a cellular process that promotes proteolytic degradations of cytosolic components at the lysosome/vacuole [26]. It has been suggested that this process is important for balancing sources of energy during development and in response to metabolic stress [27]. Under rich nutrient conditions, yeast TOR prevents the starting of the autophagy process by directly phosphorylating some of the initiation components [28].

Mitochondrial function plays a critical, albeit not completely understood, role in lifespan and stress-response determination. In this work we investigate the function of Slm35 (Yjr100c), an uncharacterized protein that has been previously found in mitochondria in large-scale proteomic studies [29, 30]. In addition, a genome-scale study led to propose a role in the biogenesis, genome maintenance, and inheritance of this organelle and therefore the gene was named *AIM25* (for Altered Inheritance of Mitochondria, [31]). In particular, it was observed that a mutant lacking this gene shows an increased loss of mtDNA when compared to a wild-type strain [31]. Since we show here that the product of YJR100C is involved in cell responses to stress and longevity, we named it *SLM35* for Stress and Longevity-related Mitochondrial factor with a predicted molecular mass of 35 kDa.

The predicted secondary structure places Slm35 within a family of phospholipid scramblases conserved in all eukaryotic cells, responsible for modulating the distribution of phospholipids within biological membranes in response to stress signals, apoptosis and mitophagy [32-34]. In humans, there are four homologous phospholipid scramblases (hPLSCRs) localized in different subcellular compartments (*e.g*. plasma membrane and mitochondria). In contrast, in *S. cerevisiae*, the mitochondrial Slm35 is the only protein that has been suggested to exhibit scramblase activity [32]; however, its function so far has remained elusive. In yeast, phospholipid metabolism is highly related to the regulation of cellular processes such as nutrient uptake and longevity [35], and a recent study showed that sphingolipid metabolism is regulated by the Tor1-Sch9 signaling pathway by a nutrient-dependent transcriptional mechanism [36].

In this study, we investigated the role of the mitochondrial protein Slm35 in stress response, lifespan and autophagy in *S. cerevisiae*. We identified a functional link with Tor1 and propose that Slm35 is involved in the crosstalk between the cytosolic stress sensing pathways and mitochondrial function through detoxification systems, mitochondrial dynamics and mitophagy.

## RESULTS

### *SLM35* encodes a non-essential mitochondrial protein that interacts genetically with *TOR1* in stress response

Inspection of the promoter region sequence of *SLM35* revealed a number of putative regulatory elements, which suggest a possible transcriptional regulation of *SLM35* during stress conditions and changes in growth rate (Supplementary Table S1). We identified three putative binding sites for transcriptional regulation factors, namely STRE, HAP and PDS. One STRE (STress Response Element) site, with the consensus sequence CCCCT, lies at −163 bp relative to the translation initiation site [37], one HAP2/3/4/5 (HAP) complex binding site at −544 bp corresponds to the consensus sequence TNATTGGT [38], and one PDS (Post-Diauxic Shift) element at −678 bp matched with the consensus T(T/A)AGGGAT [39]. All of these elements are also present in the promoter of genes encoding scavenging enzymes such as Sod2 and Ctt1, and serve to regulate its expression during stationary phase and nutrient limitation [38]. The transcriptional repression or activation of these genes is regulated by the TOR/RAS pathways through the transcription factors Msn2/4 and Gis1 in response to a wide range of stresses and nutrient availability [37, 40]. We reasoned that the presence of PDS and STRE regulatory elements in the promoter of *SLM35* might indicate the involvement of *SLM35* in the stress response and aging through these signaling pathways.

**Figure 1:**
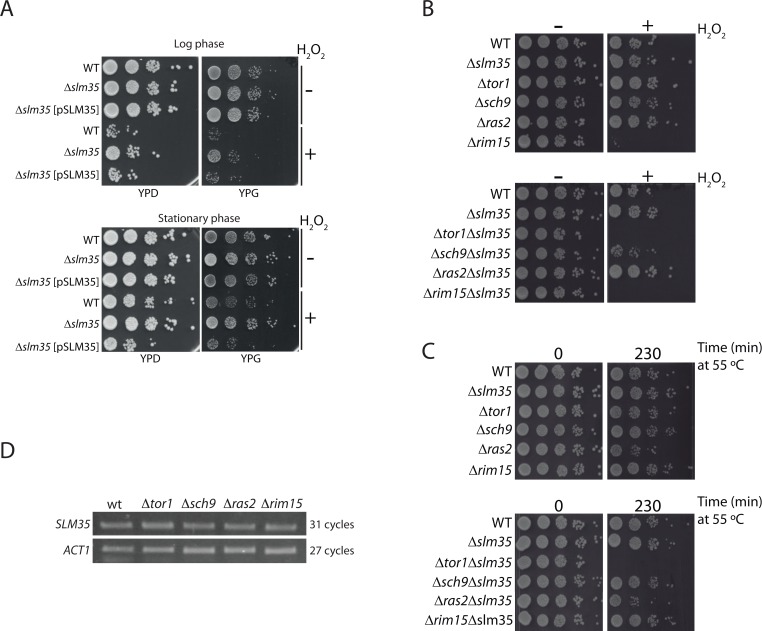
*SLM35* genetically interacts with *TOR1* during stress conditions (**A**) A strain lacking *SLM35* shows resistance to stress by hydrogen peroxide. Log phase (upper panel) or stationary phase (lower panel) cultures of a wild-type (WT) and a D*slm35* with or without a plasmid with the *SLM35* gene were treated with hydrogen peroxide (H_2_O_2_) for two hours, before serial ten-fold dilutions were dropped on rich media with glucose (YPD) or glycerol (YPG) as carbon sources. (**B, C**) Strains lacking *SLM35* (D*slm35*), components of the TOR/RAS pathways (D*tor1*, D*sch9*, D*ras2* and D*rim15*), or a combination of both, were subjected to (**B**) oxidative stress or to (**C**) heat shock as described in Materials and Methods. Serial 1:10 dilutions were dropped on rich media with glucose (YPD). (**D**) Total RNA from exponentially grown wild-type (WT), D*tor1*, D*sch9*, D*ras2* and D*rim15* strains were purified. Equal amounts of RNA were used for retro-transcription reactions and resulting cDNAs were probed with *SLM35* and *ACT1* specific primers for the indicated cycles. Reactions were visualized by denaturing electrophoresis.

*SLM35* is not an essential gene as its deletion does not elicit any observable growth phenotype under standard laboratory conditions using either fermentable or non-fermentable carbon sources (Supplementary Figure S1). To evaluate the role of Slm35 in connection with aging and stress-response genes, we studied the growth phenotype of a D*slm35* mutant under stress conditions. We evidenced that the D*slm35* strain survived better than its wild-type counterpart when stressed with hydrogen peroxide, both in fermentative (YPD) and respiratory (YPG) conditions (Figure 1A). We verified that this resistance to hydrogen peroxide-induced stress is exclusively due to the lack of *SLM35* since the addition of a plasmid expressing this gene under the control of the *GAL1* promoter (D*slm35* [pSLM35]) restored the sensitivity observed in the wild-type strain (Figure 1A). Since stress-response genes are specifically induced during the metabolic shift experienced by non-dividing cells in post-diauxic stationary phase [41], we decided to test the relevance of *SLM35* under this condition as well. Overall, cells in the stationary phase are more resistant to the hydrogen peroxide stress than those from the log phase, but in both cases the D*slm35* strain exhibits increased resistance to the stress (Figure 1A). From this we concluded that Slm35 is important for cellular stress responses during both the log and stationary growth phases.

In addition, we analyzed whether the deletion of genes involved in the nutrient-sensing TOR/RAS pathways would produce a synthetic genetic interaction with *slm35-null* mutant. For this, we selected mutants for genes involved in either of the pathways such as *TOR1*, *SCH9*, *RAS2*, and *RIM15* (Euroscarf) and deleted the entire *SLM35* open reading frame in the single mutants. We subjected these strains to either an oxidative stress with hydrogen peroxide or a heat shock stress (Figures 1B and 1C). We observed that the D*tor1*D*slm35* double mutant did not grow under either of these two stress conditions, showing a clear synthetic genetic interaction. This severe phenotype was only observed in the D*tor1*D*slm35* mutant, suggesting a specific functional relationship between *SLM35* and *TOR1*, but not with other genes involved in the TOR/RAS pathways.

The TOR/RAS signaling pathways converge on Rim15, a glucose-repressible protein kinase that regulates the expression of genes involved in stress response [23]. One possibility that could explain the functional connection found between *TOR1* and *SLM35* is that the expression of the latter would be modulated by the upstream components of these pathways. In order to address this possibility, we tested the expression of *SLM35* by semi-quantitative RT-PCR in log phase strains lacking *TOR1, SCH9, RAS2*, or *RIM15* (Figure 1D). The expression levels of *SLM35* were not affected in any of the analyzed mutants, indicating that the expression of *SLM35* during exponential growth is independent from the expression of genes of the TOR/RAS pathways.

### *SLM35* interacts genetically with the master regulator of lifespan *TOR1*

**Figure 2:**
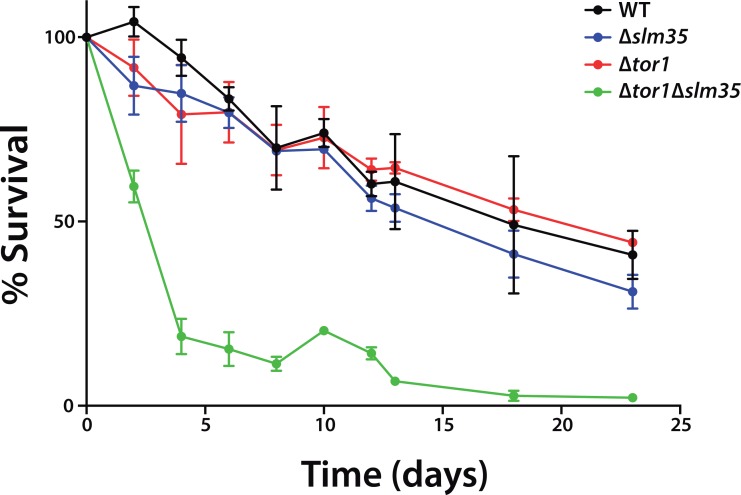
*SLM35* and *TOR1* show an aggravating genetic interaction during chronological lifespan Cells from the indicated strains were grown on SDC medium for 3 days until stationary phase. This was set as day 0, and every 2 or 3 days aliquots were taken to monitor growth as a function of time for each age point until the original culture reached 22 days. Each strain was cultured in triplicate and each aliquot was growth in duplicate. Error bars represent standard deviations.

The TOR pathway modulates the chronological and replicative lifespan of yeast. It has been previously reported that either the genetic deletion of the *TOR1* gene, or the pharmacological inhibition of *TOR1* by the macrolide antibiotic rapamycin has anti-aging effects [11, 14]. To analyze if Slm35 has a role in this process, we examined its effect on yeast CLS. Cultures from a wild-type strain and null mutants Δ*tor1*, Δ*slm35* and Δ*tor1*Δ*slm35* were grown until they reached stationary phase. This point was considered as time zero, and aliquots from each culture were taken every two or three days to analyze their growth on fresh media and to obtain age points as described previously [42]. The Δ*tor1* and Δ*slm35* strains did not show any difference in longevity compared to that of wild-type cells (Figure 2). Previous reports have shown that the sole elimination of *TOR1* promotes extension of CLS [14, 25, 41, 43], however, CLS behavior is not always the same among yeast strains and depends on the genetic background. Our results using the BY4741 wild-type background are similar to what was reported earlier [44] and probably respond to the minor respiratory capacity of this strain compared to others like W303 [45]. Interestingly, we found that the deletion of *SLM35* in combination with *TOR1* has a strong pro-aging effect. Cells from this strain significantly reduce their half-lifespan six-fold compared to the single mutants and wild-type strains (Figure 2, green line). This result suggests that the interaction of Slm35 and Tor1 is important for the regulation of cell longevity and elimination of both is detrimental for cell survival.

### Slm35 does not affect the levels of Sod2 but is necessary for catalase activity in the absence of Tor1

**Figure 3:**
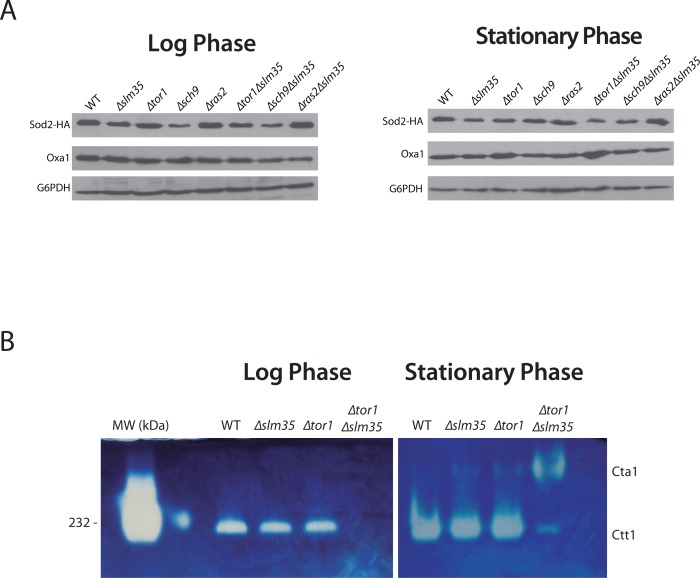
*SLM35* is involved in the regulation of the antioxidant system (**A**) Indicated strains harboring a plasmid encoding *SOD2-2HA* under the control of its endogenous promoter were grown as before using SDC-URA. 50 μg of whole cell protein extracts were analyzed by Western blot using specific antibodies to detect Sod2 (HA) or G6PDH as loading control. Signals from three independent experiments were quantified by densitometry and are shown in Supplementary Figure S2. (**B**) Determination of catalase activity in log and stationary phase of wild-type, ∆*slm35*, ∆*tor1* and ∆*tor1*∆*slm35* strains. Cells were grown on SDC medium for 14-16 h (Log phase, left panel) or 3 days (Stationary phase, right panel). 50 μg of whole cell extracts were analyzed by native gel electrophoresis and catalase activity was determined as described in Materials and Methods. Cta1, catalase A; Ctt1, catalase T. Catalase from bovine liver from (HMW Native Marker Kit, GE Healthcare) was used as molecular weight marker and catalase activity control (first lane, left panel). A representative experiment out of three is shown.

One possibility that could explain the observed sensitivity to hydrogen peroxide and heat stress as well as the pro-aging effect in the Δ*tor1*Δ*slm35* double mutant is that the antioxidant system is unable to produce scavenging enzymes. First, we analyzed the steady state levels of Sod2, a mitochondrial protein involved in cell detoxification that degrades the superoxide anion produced by the mitochondrial electron transport chain [22]. To test if *SLM35* modulates the levels of Sod2, we measured the amount of Sod2 by immunoblotting analysis in whole cell extracts (Figure 3A). The absence of *SLM35* did not impact the steady-state levels of Sod2 at log phase or stationary phase. Deletion of either *TOR1* or *RAS2* also resulted in wild-type levels of Sod2 under both conditions tested. Strains in log phase lacking *SCH9* (Δ*sch9* or Δ*sch9*Δ*slm35*) showed an apparent decrease of Sod2 steady state levels, although this decrease was only statistically significant in the Δ*sch9*Δ*slm35* strain (Supplementary Figure S2), p ≤ 0.05. Sod2 levels were unchanged in the Δ*tor1*Δ*slm35* and Δ*ras2*Δ*slm35* double mutants. The observation that Sod2 protein levels were unchanged in the Δ*tor1*Δ*slm35* strain during the log and stationary phases relative to both, the single mutant or with wild-type strains, indicates that the stress sensitivity cannot be attributed to reduced amounts of Sod2.

Next, we tested if the activity of Ctt1 and Cta1, the two catalases that are important for hydrogen peroxide scavenging [46], was affected by loss of Slm35. Catalase activity was tested using wild-type, Δ*slm35*, Δ*tor1* and Δ*tor1*Δ*slm35* whole cell extracts as described in materials and methods. The activity of Ctt1, the cytosolic catalase, in the single Δ*slm35* and Δ*tor1* mutants was similar to that of the wild-type strain in both log and stationary phase (Figure 3B). In contrast, in the Δ*tor1*Δ*slm35* mutant Ctt1 activity was completely absent (Figure 3B). When this strain was analyzed in stationary phase we could observe a low level of Ctt1 activity, and the appearance of Cta1 activity, the peroxisomal catalase, which is hardly detectable in any of the other strains analyzed. This misregulation of the antioxidant system is a possible explanation for the important decrease in longevity and stress resistance observed before in the Δ*tor1*Δ*slm35* strain.

### Slm35 alters the structure of the mitochondrial network

**Figure 4:**
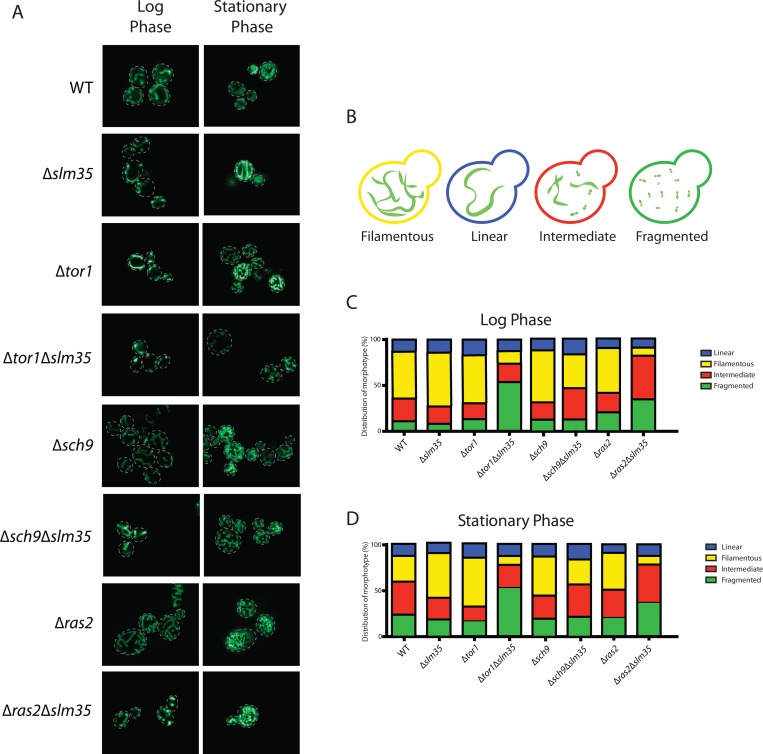
Slm35 and Tor1 or Ras2 are needed to maintain a filamentous mitochondrial network (**A**) Cells of the indicated strains harboring a plasmid encoding a mitochondrial version of GFP were grown on SDC-URA medium for 14-16 hours (Log Phase) or 3 days (Stationary Phase) and analyzed by confocal laser scanning microscopy. (**B**) The analyzed images allowed the identification of four distinct morphotypes represented here. (**C, D**) Qualitative analysis of the images presented in A as described in Materials and Methods. Log phase WT n=364; *∆slm35* n=340, *∆tor1* n=346, *∆sch9* n=360, *∆ras2* n=368, *∆tor1 ∆slm35* n=350, *∆sch9 ∆slm35* n=352, *∆ras2 ∆slm35* n=362). Stationary phase (WT n=350; *∆slm35* n=344, *∆tor1* n=349, *∆sch9* n=367, *∆ras2* n=366, *∆tor1 ∆slm35* n=353, *∆sch9 ∆slm35* n=274, *∆ras2 ∆slm35* n=365).

Studies in many organisms have shown that aging disturbs the morphology of the mitochondrial network [3, 47, 48]. Since we observed a pro-aging effect in a Δ*tor1*Δ*slm35* strain, we hypothesized that the mitochondrial network could also be affected in this mutant. We chose to evaluate the integrity of the mitochondrial network in strains lacking *SLM35* alone or in combination with the absence of *TOR1, SCH9* and *RAS2* during log and stationary phase. A plasmid encoding a mitochondrial version of GFP (pVT100U-mtGFP, [49]) was introduced into each of these strains, and the structure of the mitochondrial network was analyzed by confocal laser scanning microscopy (Figure 4A). We could identify four distinct mitochondrial morphotypes within every population: 1) linear with elongated and unbranched mitochondria; 2) filamentous with branched mitochondria; 3) an intermediate morphotype between fused and fragmented mitochondria; and 4) fragmented with punctate mitochondria (Figure 4B).

Under normal growth conditions the wild-type strain showed a higher proportion of filamentous branched mitochondria. The Δ*slm35*, Δ*tor1* and Δ*sch9* single mutants presented a slight increase in filamentous mitochondria compared to wild-type under these conditions, and there was no apparent difference between the Δ*ras2* and the wild-type. Strikingly, in Δ*tor1*Δ*slm35* and Δ*ras2*Δ*slm35* double mutants, the filamentous phenotype was severely reduced, in these mutants the fragmented or intermediate morphotypes, respectively, were more frequent (Figure 4A and C). During the stationary phase, wild-type mitochondria presented a lower filamentous morphotype and the intermediate and fragmented ones increased. The Δ*slm35*, Δ*tor1* and Δ*sch9* single mutants showed an increase in filamentous mitochondria compared to the wild-type, in a similar fashion as it was observed in the log phase. The Δ*tor1*Δ*slm35* and Δ*ras2*Δ*slm35* double mutants presented the same phenotype observed in log phase, where filamentous mitochondria were severely reduced (Figure 4A and D). These results showed that Slm35 is necessary to maintain the integrity of the mitochondrial network in both log and stationary phase.

### *SLM35* genetically interacts with autophagy-related genes and regulates mitophagy flux

**Figure 5:**
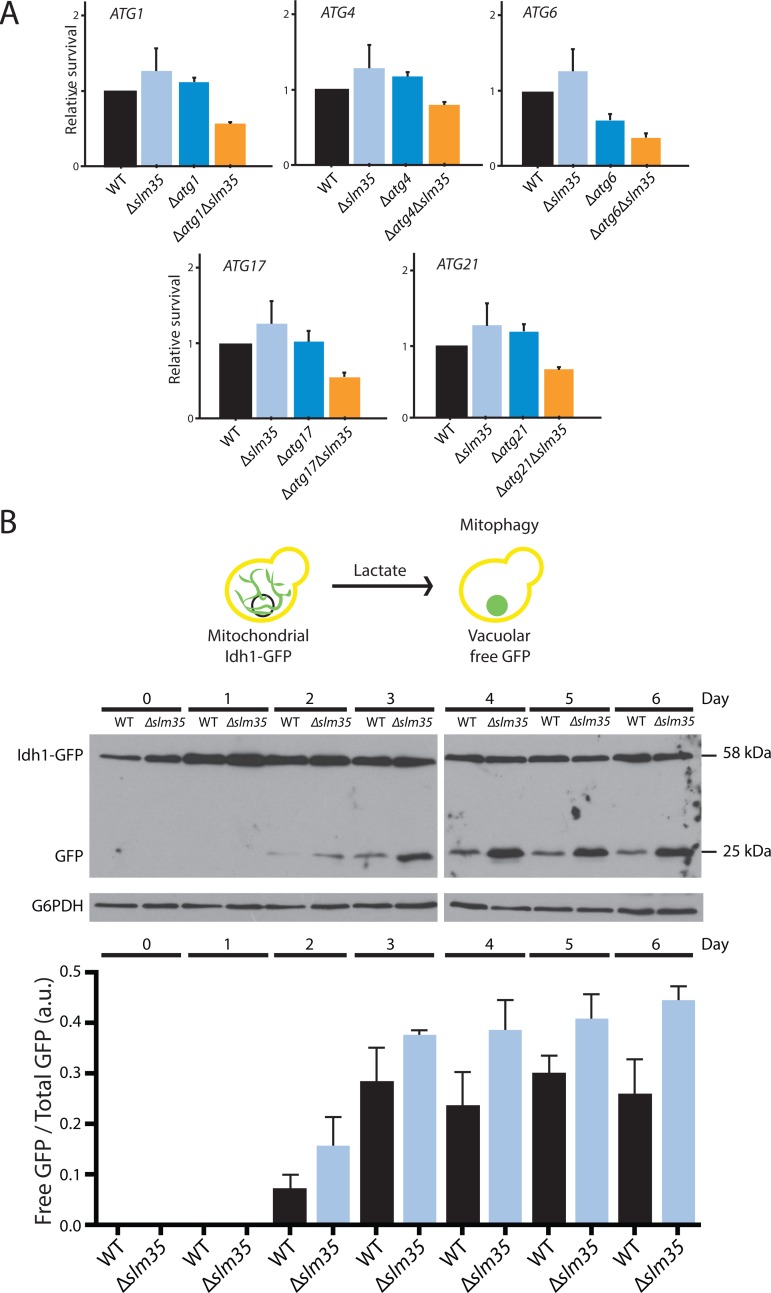
The function of Slm35 is necessary for the regulation of general and mitochondrial selective autophagy (**A**) Relative survival under heat shock conditions of wild-type (WT), single mutants Δ*slm35*, Δ*atg1*, Δ*atg4*, Δ*atg6,* Δ*atg17*, and Δ*atg21*, or double mutants Δ*atg1*Δ*slm35*, Δ*atg4*Δ*slm35*, Δ*atg6*Δ*slm35*, Δ*atg17*Δ*slm35*, and Δ*atg21*Δ*slm35.* Cells were grown on SCD liquid medium during three days before applying a thermal shock of 20 min at 55°C. Aliquots were taken immediately after the stress to construct YPD growth curves to calculate the relative survival. (**B**) Overnight YPD cultures of wild-type or Δ*slm35* cells harboring the fusion protein Idh1-GFP were shifted to YPL medium to an OD_600_=0.1 to induce mitophagy. Every 24 hours an aliquot of the culture was taken and whole cell extracts were analyzed by Western blot. Mitochondrial Idh1-GFP runs as a discrete band at around 60 kDa, vacuolar free GFP runs at 25 kDa. G6PDH was used as loading control. Signals from three independent experiments were quantified by densitometry.

Autophagy is a cytoprotective mechanism that plays an integral role in the eukaryotic stress response [50, 51], and Tor1 regulates autophagy in response to nutrient availability and stress conditions [28]. To determine whether Slm35 directly participates in the autophagy pathway, we performed a genetic analysis under heat shock conditions (55°C). As shown in Figure 5A, *SLM35* showed a negative genetic interaction with some of the genes that participate in the autophagy response, namely *ATG1*, *ATG4*, *ATG6*, *ATG17,* and *ATG21*. The deletion of *SLM35* or any of the autophagy-related genes (Atg) showed no effect on the survival of yeast cells after heat shock; however, the elimination of *SLM35* in combination with the aforementioned genes, significantly decreased the survival below the wild-type value. This led us to assume that Slm35 and autophagy are functionally related during cell response to heat stress.

These results suggested that Slm35 is involved in the regulation of autophagy to control mitochondrial morphology and cell resistance to stress. Upon starvation or cellular damage conditions, mechanisms such as mitochondrial selective autophagy or mitophagy are activated to avoid cell death [52, 53]. To determine the potential contribution of Slm35 in this specific type of autophagy, we deleted the entire *SLM35* open reading frame in the strain EY0986, where the mitochondrial protein Idh1 is fused to GFP (Idh1-GFP [54]). Mitophagy was induced as previously reported [55], by shifting the cell culture from rich media with glucose (YPD) to rich media with lactate (YPL) and the mitophagic flux was monitored by the release of free GFP that results from vacuolar processing. The vacuolar form of GFP could be observed after three days of growth in YPL in both wild-type and Δ*slm35* strains; however, the absence of Slm35 resulted in an increased mitophagy flux compared to wild-type strain (Figure 5B). This data confirmed a role for Slm35 as a negative regulator of mitophagy in yeast, although at this point we cannot discard if this effect is direct or indirect.

## DISCUSSION

Pathways that regulate nutrient uptake, stress resistance and longevity are well studied but the role of mitochondria within these processes is not completely understood. This study shows that the mitochondrial protein Slm35 is an important player in these processes, regulating longevity, stress response, mitochondrial morphology and homeostasis, mainly through the TOR pathway.

*SLM35* deletion is characterized by a resistance growth phenotype under oxidative or heat shock, similar to what was previously reported for ∆*tor1*, ∆*sch9* and ∆*ras2* mutants [23, 43]. Strikingly, the simultaneous deletion of *SLM35* and *TOR1* completely abolished the cytoprotective responses of the cell under stress conditions. This hypothesis is strengthened by the observation that the CLS of a ∆*tor1*∆*slm35* double mutant is severely compromised. On the other hand, deletion of *SLM35* had no effect on the stress resistance of ∆*ras2* mutants, and had only a mild effect on ∆*sch9* strains, supporting the idea that Slm35 exerts its function through Tor1. It could be possible to assume that the deletion of *SLM35* prevents the responses triggered in a ∆*tor1* strain, for example by deregulating Rim15 and the downstream transcription factors Msn2/4 previously related to the TOR pathway [13, 56, 57].

The control of superoxide toxicity plays an important role during aging and cell death [58]. In a ∆*tor1* mutant the expression of *SOD2* increases during late stationary phase and contributes to stress resistance, but not to lifespan extension [41]. Contrary, Sod2 is required for survival extension in a ∆*sch9* strain [58]. Curiously, we found that the levels of the protein Sod2 were not modified neither during log nor stationary phases in the double ∆*tor1*∆*slm35*. Our data suggests that Sod2 is dispensable for lifespan extension and stress response in this strain. In addition to Sod2, catalase is an important hydrogen peroxide scavenging enzyme that participates in ROS homeostasis and whose function has been associated to modifications in the CLS [59-62]. The lower activity of Ctt1 observed in a ∆*tor1*∆*slm35* could explain the observed sensitivity to hydrogen peroxide in this strain. The effect of catalase on CLS is highly variable, and depends on the growth substrates [60]. Some reports show that deletion of *CTA1* (gene encoding the catalase present in peroxisomes and mitochondria) causes a decrease in CLS [59], while others report that *CTA1* or *CTT1* elimination increase the CLS by inducing elevated levels of ROS which induce SOD activity [62]. Our data revealed that the Cta1 activity increased in ∆*tor1*∆*slm35* in stationary phase. This stimulation probably reduces the intracellular levels of hydrogen peroxide needed to activate responses for survival thereby compromising cell viability, similar to what it was reported for Cta1 overexpression [62].

Loss in volume and increase of mitochondrial fragmentation has been associated with aging [48, 63]. Furthermore, mitochondrial morphology is dynamic and responds to changes in cell metabolism, balance between mitochondrial fission and fusion and nutrient sensing mechanisms involving the TOR pathway [64]. In agreement with this idea, we observed that in a wild-type strain there is a shift from filamentous mitochondria to a more fragmented phenotype as cells age. Our results suggest that the elimination of *SLM35* could preserve the mitochondrial morphology in a similar way as the *TOR1* deletion. Interestingly, we observed that in stationary phase, the double mutant ∆*tor1*∆*slm35* notably increased the proportion of fragmented mitochondria. This behavior could be a direct consequence of the short-lived phenotype observed as it has been proposed before [48], and suggests that the concomitant elimination of *SLM35* and *TOR1* has an effect on mitochondrial biogenesis.

Heat shock conditions compromise the homeostatic state of the cell, mainly by altering protein folding [65]. To preserve protein homeostasis under stress conditions, autophagy plays a crucial role by limiting the accumulation of protein aggregates and damaged mitochondria, maintaining sufficient energy levels to survive stressful conditions [66]. In the present study, we demonstrated that *SLM35* has aggravating genetic interactions under thermal conditions with genes involved in autophagy mechanisms, denoting a possible participation of *SLM35* during early stages of autophagosome formation [67].

We also evaluated mitophagy, a particular form of selective autophagy that specifically degrades mitochondria. During growth on lactate as carbon source, the metabolism of yeast changes from fermentative to respiratory, and although initially the amount of mitochondria increases to fulfill the energetic requirements, cells age earlier and promote mitophagy to regulate the amount of mitochondria [55]. Mitophagy flux in ∆*slm35* mutant was significantly increased compared to the wild-type, indicating a role for Slm35 as a negative regulator of mitophagy.

We hypothesize that under normal growth conditions, Slm35 triggers signaling that reach the autophagy core assembly, like Atg1 and Atg17, similar to what has been previously reported for Tor1 [28, 68]. Furthermore, it has been reported that the inhibition of Tor1 in yeast maintains the mitochondrial structure in an autophagy-dependent manner by controlling the phosphorylation of Atg13 and the consequent binding of Atg1 [28]. The presence of Slm35 can inhibit autophagy initiation as a mechanism to regulate mitochondrial quality control, hence its elimination promotes an upregulation of the mitophagic flux. Another possibility is that since mitophagy occurs more efficiently in the absence of Slm35 any portion of the mitochondrial network could be promptly removed, preserving the filamentous mitochondrial network and improving cellular fitness under stress conditions.

Although the precise mechanism of action of Slm35 remains largely uncharacterized, it could function rearranging phospholipids within the mitochondrial membranes in response to stress signals as it has been previously reported for its mammalian homologue, human phospholipid scramblase 3 (hPLSCR3), which mediates the apoptotic response [33], or the externalization of cardiolipin to the outer membrane as a signal for mitophagy [34]. In yeast, the relationship between autophagy, lipid homeostasis and longevity has also been recently reported [44]. Our results suggest that Slm35 could constitute a functional bridge between the cytosolic information initiated by stress signals or nutrient deficiencies through the TOR pathway and mitochondrial responses.

## MATERIALS AND METHODS

### Strains and plasmids

All the *S. cerevisiae* strains used in this study are isogenic to the S288C-derivative BY4741 or EY0986 [54, 69] (Supplementary Table S2). Knockout strains were generated by homologous recombination using specific primers designed to replace the entire open reading frames by the *kan*MX4 cassette amplified from the pFA6a plasmid [70]. Each replacement was verified by PCR. To express *SLM35* in yeast cells the complete ORF YJR100C was cloned into the plasmid pYES2.0 (Invitrogen) using the primers SLM35-EcoRI F (5′- GAAGAATTCATGCATAGAACGGCAATATTTC-3′) and SLM35-XhoI R (5′- GTTCTCGAGCTACTCATCATAGCCACCG -3′). *SOD2*-HA was cloned into the plasmid pRS316 [71] with the addition of two hemagglutinin tags under the regulation of its endogenous promoter.

### Growth conditions

Yeast cultures were grown at 30°C on rich YP (yeast extract 1%, bactopeptone 2%), synthetic S (yeast nitrogen base without amino acids and ammonium sulfate 1.7 g/L, (NH_4_)_2_SO_4_ 5 g/L; with all supplements SC or defined without uracil SD-URA); using either glucose 2% w/v, lactate 2%, or glycerol 2% [72, 73].

### Chronological lifespan assay

Yeast chronological lifespan was measured based as previously described [42]. Briefly, over-night cultures in SCD 2% medium were diluted 1:2000 in 10 mL of fresh medium and further incubated for 22 days at 30°C shaking at 250 rpm. Starting three days later, age points were taken every 2 days. At each age point, 10 μL were taken and diluted 1:30 with YPD to start growth curves using an automated microbiology growth curve analysis system Bioscreen C at 30°C and continuous shaking. Data analysis was performed according to the considerations set by Murakami and Kaeberlein [42].

### Stress resistance assays

Resistance to oxidative stress was measured by growth after a shock with hydrogen peroxide. Cells were cultured in liquid media to growth or stationary phase as indicated. Cultures were then diluted to an OD_600_ of 1.0 in phosphate buffer (K_2_HPO_4_/KH_2_PO_4_) 10 mM pH 6.0, and treated with 350 mM hydrogen peroxide (Sigma-Aldrich) for 60 or 120 min. Serial 1:10 dilutions from each culture and a non-treated control were spotted onto YPD or YPG plates and incubated at 30°C for 2 or 3 days respectively. Resistance to temperature stress was measured by growth after a heat-shock at 55°C. Cells were cultured in liquid media to log or stationary phase as indicated. Cultures were then diluted to an OD_600_ of 1.0 in phosphate buffer (K_2_HPO_4_/KH_2_PO_4_) 10 mM pH 6.0, and serial 1:10 dilutions from each culture were spotted onto YPD or YPG plates. Plates were incubated at 55°C (heat-shock) for 200 and 245 min before further incubation at 30°C for 2 or 3 days.

### RNA extraction and RT-PCR

Each strain was grown in the appropriate medium until an OD_600_ of 2.0 was reached. 20 mg of wet weight cells were collected by centrifugation 5 min at 16,873*xg*. The cell pellet was shock frozen in liquid nitrogen and ground using a porcelain mortar and pestle. Total RNA was extracted using Trizol (Ambion) following the manufacturer's recommendations. DNase-treated RNA (300 ng) was then reverse-transcribed in a total volume of 20 μL using the ProtoScript M-MuLV First Strand cDNA Synthesis Kit using the oligo-dT primer provided by the manufacturer (New England Biolabs).

### Catalase activity assay

Lysate protein for enzymatic determination were obtained from cell disruption, 7 cycles of 1 min of vortexing followed by 1 min of cooling on ice, with glass beads in 50 mM KPi (pH 7.0), 1 mM PMSF, 1 mM EDTA buffer [74]. Protein concentration was determined by the method of Bradford (Biorad) using BSA as standard and 50 μg of lysate protein were analyzed on 8% clear native gels. Catalase activity was determined as previously described [75]. The native gel was incubated in a 0.01% hydrogen peroxide solution for 5 min, rinsed with water, and incubated with 2% w/v FeCl_3_ and 2% w/v K_3_[Fe(CN)_6_] solution until the formation of a blue precipitate and the appearance of transparent bands on the gel.

### Analysis of mitochondrial morphology by confocal microscopy

Strains were transformed with the plasmid pVT100U-mtGFP [49] and grown on SCD without uracil medium during 14 h for log phase, or 3 days for stationary phase. Cells were transferred onto a slide, mixed with glycerol 100% and visualized using an FV10i confocal microscope (Olympus) with a water immersion objective (60X). Mitochondrial morphotypes were identified as previously reported [47] and manually quantified.

### Mitophagy assay

Wild-type and D*slm35* strains expressing the mitochondrial protein Idh1 tagged with GFP [54] were grown in YPD until mid-log phase and diluted into YPL (OD_600_=0.1) to induce mitophagy as previously described [55]. Cells corresponding to 2.0 OD 600 nm were collected by centrifugation every 24 hours and whole cell extracts were prepared by lysing the cells with a buffer containing 0.3 N sodium hydroxide, 176 mM β-mercaptoethanol and 3.5 mM phenylmethyl-sulfonyl fluoride and incubating them 10 min on wet ice. Proteins were then precipitated using 12% w/v trichloroacetic acid and washed with cold acetone. The resulting protein pellet was resuspended in 2% SDS and the protein content was quantified by Bradford (BioRad). Fifty micrograms of protein were loaded on 17.5% SDS-polyacrylamide gels and separated proteins were electrotransferred onto nitrocellulose membranes and subsequently decorated with a specific antibody against GFP.

### Miscellaneous

DNA manipulations were carried out according to standard procedures and all the resulting constructs were verified by sequencing. Antibodies used in this study were purchased from LifeSpan BioSciences, Inc. (Polyclonal anti-GFP, peroxidase conjugated), Thermo Scientific (goat anti-rabbit IgG, F(ab’)_2_, peroxidase conjugated), Roche (monoclonal rat anti-HA-peroxidase), and Sigma-Aldrich (monoclonal rabbit anti- Glucose-6-Phosphate Dehydrogenase (G-6-PDH)). Oxa1 antibody was a kind gift from Johannes M. Herrmann (U. Kaiserslautern, Germany). All statistical analyses were performed using Prism 6.0.

## SUPPLEMENTARY FIGURES AND TABLES



## Abbreviations

ROS: Reactive oxygen species
CLS: Chronological Lifespan
