# Supplementary material for: Slm35 links mitochondrial stress response and longevity through TOR signaling pathway
Source: Aging (Albany NY). 2016 Dec 2;8(12):3255–68. doi: 10.18632/aging.101093 (PMC5270667; doi:10.18632/aging.101093)
Supplement: Supplementary file 1 [file aging-08-3255-s001.pdf]

SUPPLEMENTARY MATERIAL

Supplementary Table S1. Putative *SLM35* promoter regulation sites

Putative Element	Consensus	Name
-163 CCCCT -159	CCCCT	STRE
- 544 TGATTGGT -536	TNATTGGT	HAPA
-678 TAAGGGAT -670	T(T/A)AGGGAT	PDS

Supplementary Table S2. List of strains used in this study

Strain name	Genetic background	Genotype	Reference
WT	BY4741	*MATa his3Δ1 leu2Δ0 met15Δ0 ura3Δ0*	Brachmann et al. 1998
□*slm35*	BY4741	*MATa his3Δ1 leu2Δ0 met15Δ0 ura3Δ0 slm35::KanMX*	Euroscarf
□*tor1*	BY4741	*MATa his3Δ1 leu2Δ0 met15Δ0 ura3Δ0 tor1::KanMX*	Euroscarf
□*sch9*	BY4741	*MATa his3Δ1 leu2Δ0 met15Δ0 ura3Δ0 sch9::KanMX*	This work
□*ras2*	BY4741	*MATa his3Δ1 leu2Δ0 met15Δ0 ura3Δ0 ras2::KanMX*	Euroscarf
□*rim15*	BY4741	*MATa his3Δ1 leu2Δ0 met15Δ0 ura3Δ0 rim15::KanMX*	Euroscarf
□*tor1* □*slm35*	BY4741	*MATa his3Δ1 leu2Δ0 met15Δ0 ura3Δ0 tor1::KanMX slm35::hph*	This work
□*sch9* □*slm35*	BY4741	*MATa his3Δ1 leu2Δ0 met15Δ0 ura3Δ0 sch9::KanMX slm35::hph*	This work
□*ras2* □*slm35*	BY4741	*MATa his3Δ1 leu2Δ0 met15Δ0 ura3Δ0 ras2::KanMX slm35::hph*	This work
□*rim15* □*slm35*	BY4741	*MATa his3Δ1 leu2Δ0 met15Δ0 ura3Δ0 rim15::KanMX slm35::hph*	This work
Δ*atg1*	BY4741 x Y8205	MATa PDC1-mCherry-CaURA3MX4 can1Δ::STE2pr-SpHIS5 lyp1Δ ura3Δ0 his3:kanMX4 *LEU2 MET15* atg1::natMX4	This work
Δ*atg4*	BY4741 x Y8205	MATa PDC1-mCherry-CaURA3MX4 can1Δ::STE2pr-SpHIS5 lyp1Δ ura3Δ0 *LEU2 MET15* atg4::natMX4 his3::kanMX4	This work
Δ*atg6*	BY4741 x Y8205	MATa PDC1-mCherry-CaURA3MX4 can1Δ::STE2pr-SpHIS5 lyp1Δ ura3Δ0 *LEU2 MET15* atg6::natMX4 his3::kanMX4	This work
Δ*atg17*	BY4741 x Y8205	MATa PDC1-mCherry-CaURA3MX4 can1Δ::STE2pr-SpHIS5 lyp1Δ ura3Δ0 *LEU2 MET15* atg17::natMX4 his3::kanMX4	This work
Δ*atg21*	BY4741 x Y8205	MATa PDC1-mCherry-CaURA3MX4 can1Δ::STE2pr-SpHIS5 lyp1Δ ura3Δ0 *LEU2 MET15* atg21::natMX4 his3::kanMX4	This work

Δ*slm35*	BY4741 x Y8205	MATa PDC1-mCherry-CaURA3MX4 can1Δ::STE2pr-SpHIS5 lyp1Δ ura3Δ0 *LEU2 MET15* slm35::natMX4 his3::kanMX4	This work
Δ*atg1* Δ*slm35*	BY4741 x Y8205	MATa PDC1-mCherry-CaURA3MX4 can1Δ::STE2pr-SpHIS5 lyp1Δ ura3Δ0 his3Δ1 *LEU2 MET15* atg1::natMX4 slm35::kanMX4	This work
Δ*atg4* Δ*slm35*	BY4741 x Y8205	MATa PDC1-mCherry-CaURA3MX4 can1Δ::STE2pr-SpHIS5 lyp1Δ ura3Δ0 his3Δ1 *LEU2 MET15* atg4::natMX4 slm35::kanMX4	This work
Δ*atg6* Δ*slm35*	BY4741 x Y8205	MATa PDC1-mCherry-CaURA3MX4 can1Δ::STE2pr-SpHIS5 lyp1Δ ura3Δ 0 his3Δ1 LEU2 MET15 atg6::natMX4 slm35::kanMX4	This work
Δ*atg17* Δ*slm35*	BY4741 x Y8205	MATa PDC1-mCherry-CaURA3MX4 can1Δ::STE2pr-SpHIS5 lyp1Δ ura3Δ0 his3Δ1 LEU2 MET15 atg17::natMX4 slm35::kanMX4	This work
Δ*atg21* Δ*slm35*	BY4741 x Y8205	MATa PDC1-mCherry-CaURA3MX4 can1Δ::STE2pr-SpHIS5 lyp1Δ ura3Δ0 his3Δ 1 LEU2 MET15 atg21::natMX4 slm35::kanMX4	This work
Idh1-GFP	EY0986	*MATa his3Δ1 leu2Δ0 met15Δ0 IDH1-GFP::HIS3 ura3Δ0* (S288C)	Hu et al. 2003
□*slm35* Idh1-GFP	EY0986	*MATa his3Δ1 leu2Δ0 met15Δ0 IDH1-GFP::HIS3 ura3Δ0* (S288C) slm35*::KanMX*	This work

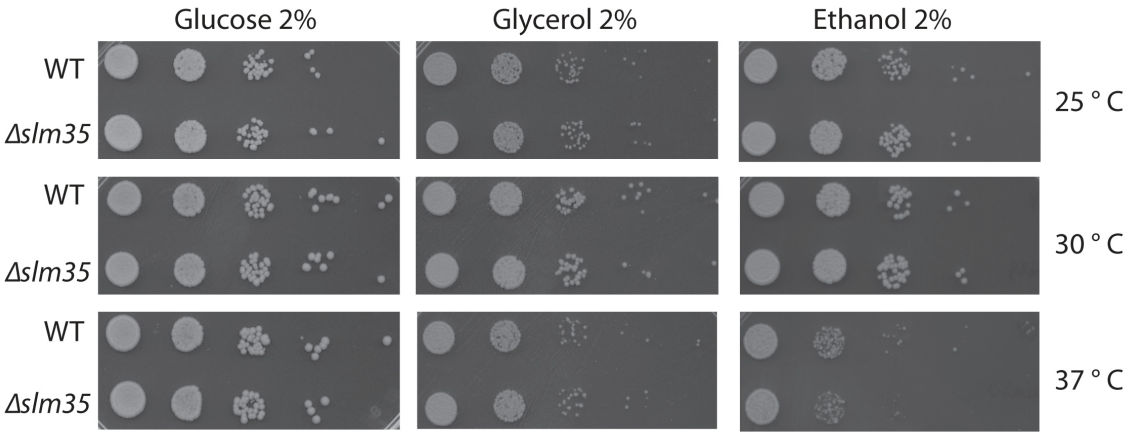

Supplementary Figure S1. Deletion of *SLM35* does not produce any observable phenotype under standard laboratory growth conditions. Wild-type and *Δslm35* strains were grown on rich media with fermentable (Glucose) and non-fermentable (Glycerol and Ethanol) carbon sources at different temperatures as indicated. Ten-fold dilutions from liquid cultures grown at 30 ^{o}C were dropped on solid medium and strains were incubated at 25, 20 and 37 ^{o}C as indicated.

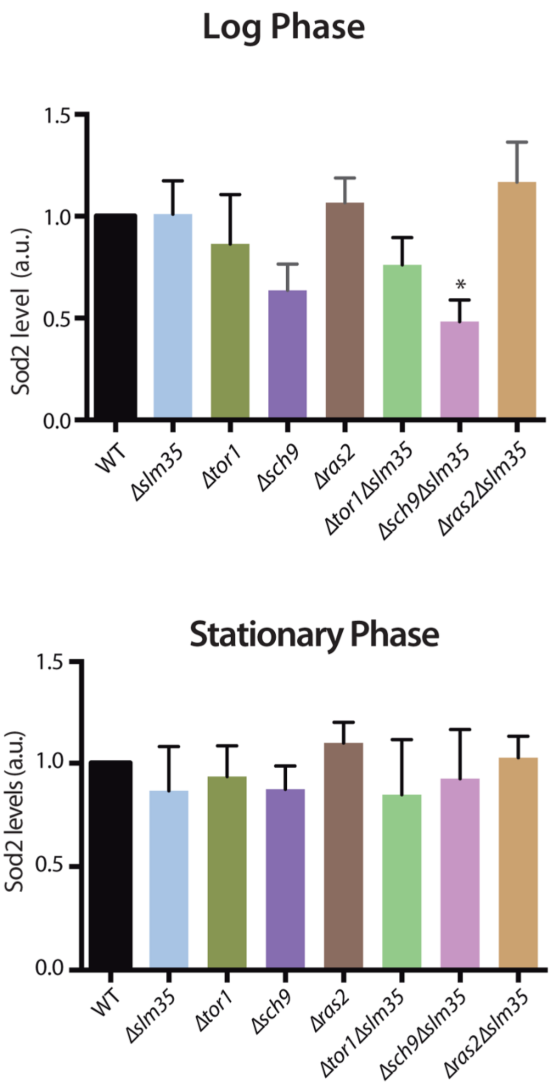

Supplementary Figure S2. The absence of *SLM35* does not compromise the amount of Sod2 present in whole cells. Quantification by densitometry of the Sod2 endogenous levels obtained in three independent experiments as shown in Figure 3. The data was normalized with the wild-type values (WT=1.0) and analyzed with an ordinary one-way ANOVA test, $p \leq 0.05$.
